# Identification and characterization of nine *PAT1* genes subfamily in *Medicago edgeworthii*

**DOI:** 10.1080/15592324.2025.2527380

**Published:** 2025-07-01

**Authors:** Gaoping Tang, Tingting Ni, García-Caparrós Pedro, Li-Hua Meng, Xudong Sun

**Affiliations:** a School of Life Sciences, Key Laboratory of Yunnan for Biomass Energy and Biotechnology of Environment, Yunnan Normal University, Kunming, Yunan, China; b Yunnan Key Laboratory of Crop Wild Relatives Omics, The Germplasm Bank of Wild Species, Institute of Tibetan Plateau Research at Kunming, Kunming Institute of Botany, Chinese Academy of Sciences, Kunming, Yunnan, China; c Higher Engineering School, University of Almeria, Almeria, Spain

**Keywords:** GRAS gene family, PAT1 subfamily gene, phylogenetic analysis, phytochrome

## Abstract

GRAS proteins represent a unique class of transcription factors that are exclusive to plants. Among the various subfamilies within the GRAS family, the phytochrome A signal transduction 1 (PAT1) subfamily is particularly prominent, given its multifaceted regulatory functions in phytochrome signaling pathways and stress response mechanisms, as well as its involvement in plant developmental processes. Despite the recognized importance of *GRAS* proteins, there are no studies to date that have characterized the *GRAS* gene family in *Medicago edgeworthii*. In this study, we performed a comprehensive genome-wide analysis of *GRAS* genes and identified nine genes belonging to the *PAT1* subfamily in *M. edgeworthii*. Multiple sequence alignment of these proteins revealed the presence of a conserved C-terminal GRAS domain, alongside a highly variable N-terminal region. Additionally, we observed that members of the *PAT1* subfamily were expressed in roots, stems, and leaves, indicating their broad involvement in the development of various tissues in *M. edgeworthii*. Furthermore, functional analysis indicated that PAT1 subfamily proteins in *M. edgeworthii* activated the expression of *MeDOF3.4* gene, indicating that PAT1 subfamily proteins may be associated with the promotion of cell proliferation and graft fusion. In conclusion, this study provided the first comprehensive characterization of *PAT1* subfamily genes in *M. edgeworthii*, establishing a foundation for future research on the functional roles of *MeGRAS* genes and providing a theoretical basis for the development of high-quality *Medicago* varieties.

## Introduction

The GRAS protein family comprises a group of plant-specific transcription factors, named after its three originally identified family members Gibberellin acid insensitive (GAI), Repressor of GA1 (RGA) and SCARECROW (SCR).^1–4^ Typical GRAS proteins usually comprise 400 ~ 770 amino acid residues and are characterized by a highly conserved C-terminal region and a variable N-terminal region.^5^ The highly conserved C-terminus of GRAS proteins can be divided into five different motifs based on their amino acid compositions:, LHR I, VHIID, LHR II, PFYRE, RVER, and SAW.^6^ Among these, the VHIID conserved sequence is the core structure of the GRAS proteins and is present across all members of the family.^7^ According to different protein structural characteristics, the GRAS family is further categorized into eight subfamilies: DELLA, LAS, SCR, SHR, PAT1, HAM, LISCL, and SCL3.^8–10^

The PAT1 subfamily is a subgroup within the GRAS transcription factor families.^2,11^ There are six PAT1 subfamily members identified in Arabidopsis, including *AtPAT1*, *AtSCL1*, *AtSCL5*, *AtSCL8*, *AtSCL13*, and *AtSCL21*.^12^ Among these, *AtPAT1*, *AtSCL5*, *AtSCL13* and *AtSCL21* are highly homologous and play important roles in regulating the phytochrome signal transduction,^13–15^ responding to abiotic stress^16–18^ and maintaining meristematic tissues.^19,20^ Specifically, *AtPAT1*, *AtSCL5*, and *AtSCL21* are positive regulators in the phytochrome A signaling pathway,^11,14^ and they share a common EAISRRDL protein motif at their N terminus, a feature which is absent in other members of the family. Another member of the PAT1 subfamily, SCL13, acts as a positive regulator in the red light signaling pathway downstream of phytochrome B,^15^ playing an essential role in the elongation of hypocotyl. Moreover, members of the PAT1 subfamily have been implicated in wound healing during grafting. PAT1 has been identified as a key regulator of graft healing in both *Picea abies* and *Arabidopsis thaliana*. Notably, *Arabidopsis thaliana* mutants lacking the *PAT1* gene could not be successfully grafted,^20^ whereas*AtPAT1* over-expressing leads to increased callus formation at the site of petiole injury.^20^ Additionally, the PAT1-ERF115 protein complex is pivotal for maintaining the function of meristematic tissues by promoting the regeneration of damaged stem cells.^21^

The DNA-binding with one finger (Dof) family represents a unique class of transcription factors exclusive to plants, belonging to the zinc finger protein subfamily.^22^ Among them, the DOF3.4 transcription factor (also known as OBP1) in *Arabidopsis thaliana* plays a crucial role in cell cycle regulation.^23^ Notably, *DOF3.4* expression is strongly activated in response to mechanical injury, highlighting its role in wound-induced signaling pathways.^19^ Consequently, *DOF3.4* gene is a crucial component driving cell division during plant regeneration. Recent studies have shown that PAT1 subfamily proteins can promote root tip regeneration in *Arabidopsis thaliana* by directly activating DOF3.4.^19^

*Medicago edgeworthii* is a perennial herbaceous species belonging to the Fabaceae family, which mainly grows in high-altitude areas such as southeastern Xizang, southern Qinghai, and northwestern Yunnan in China.^24,25^ This species is notable not only for its high nutritional value but also for its superior adaptability to the climatic conditions of these areas, attributed to its high cold tolerance.^13^ Consequently, *M. edgeworthii* can be used as one of the wild high-protein pasture germplasm resources in China’s alpine grassland,^26^ and participate in the improvement, restoration and reconstruction of these fragile ecosystems. Research has shown that polyploid plants have significant advantages over diploids in terms of biomass and stress resistance. *Medicago edgeworthii* is naturally diploid (2n = 2x = 16) and can generate new *Medicago* germplasm resources through chromosome duplication.^13^ Therefore, creating a new germplasm of allopolyploid *Medicago* by grafting and fusing it with other *Medicago* species represents a promising strategy for broadening the genetic base and enhancing the agronomic traits of this species.

In this study, we identified 58 *GRAS* genes within the complete genome of *M. edgeworthii*. Comprehensive analyses were conducted to characterize their gene structure, amino acid composition, protein physicochemical properties, protein conserved motifs, chromosomal localization, and evolutionary relationships. The *GRAS* gene family members in *M. edgeworthii* were divided into nine subfamilies with GRAS proteins clustered within the same subfamily displaying conserved motifs. Here, we cloned nine putative PAT1 subfamily genes from an *M. edgeworthii*. A sequence alignment and phylogenetic analysis further confirmed their classification within the PAT1 subfamily, and these genes have been designated as MeGRASs. Subcellular localization experiments showed that PAT1 subfamily proteins were located in the nucleus and function as a nuclear protein. Furthermore, transient expression analysis in tobacco showed that PAT1 subfamily proteins activated the expression of *MeDOF3.4* gene, indicating that PAT1 subfamily proteins might be related to promoting cell proliferation and regeneration. Collectively, these findings provided a theoretical basis for further elucidating the physiological and biochemical functions of the *GRAS* gene family in *M. edgeworthii*, thereby laying a foundation for the genetic improvement and cultivation of high-quality alfalfa varieties in the future.

## Manuscript formatting

### Materials and methods

#### Plant materials and growth conditions

All plantlets of *M. edgeworthii* were cultivated on Murashige & Skoog basal medium with vitamins (Phyto Technology Laboratories, M519) supplemented with 3.0% (w/v) sucrose and 0.8% (w/v) agar (pH 5.8) at temperatures of 25/18°C under a 16-h light/8-h dark photoperiod. To investigate the expression levels of PAT1 subfamily genes in different tissues of *M. edgeworthii*, we collected roots, stems, and leaves from 14-day-old *M. edgeworthii* plantlets. The collected materials were immediately frozen in liquid nitrogen and stored at −80℃ until RNA extraction.

#### Extraction of DNA and RNA

Total RNA was extracted according to the instructions provided with the Eastep ® Super Total RNA Extraction Kit (Promega, Madison, WI, USA). The quality of the extracted RNA was assessed using 1% agarose gel electrophoresis. Qualified RNA samples were then reverse transcribed into cDNA following the protocols of the 5X All-In-One RT MasterMix (with AccuRT Genomic DNA Removal Kit).

#### Identification of GRAS gene family members and analysis of basic physicochemical properties in M. edgeworthii

To identify GRAS family members in *M. edgeworthii*, GRAS protein sequences of *M. truncatula* and *Arabidopsis thaliana* were retrieved from the Plant Transcription Factor Database (http://planttfdb.gao-lab.org) and the Arabidopsis Information Resource (TAIR) database (http://www.Arabidopsis.org), respectively. The genome sequence data for *M. edgeworthii* are unpublished data generated in our laboratory. Using TBtools software,^27^ based on the known GRAS transcription factor family sequence of *M. truncatula*, a local BLAST search was performed on the sequence of *M. edgeworthii* to preliminarily determine the GRAS members in *M. edgeworthii* (Parameters were E < 1e^−10^, Identity > 40%). Afterwards, candidate genes were validated using the Hidden Markov Model (HMM) configuration file (login number: PF03514) obtained from the Pfam database (http://pfam.sanger.ac.uk).^28^ Additionally, the SMART tool was used to confirm the presence of conserved homologous domains within the candidate genes. Protein sequences containing GRAS typical domains (SAW, PFYRE, Leucine Heptad Repeat II (LHR II), VHIID, Leucine Heptad Repeat I (LHR I) from C-terminus to N-terminus) were screened. Genes lacking these conserved domains were excluded for further analysis, while sequences with homologous domains were retained for subsequent investigation.

Based on phylogenetic clustering with GRAS proteins of *Arabidopsis* and *M. truncatula*, the identified GRAS proteins in *M. edgeworthii* were designated as MeGRAS proteins. The ExPASy server^29^ (https://web.expasy.org/protparam/) was used to calculate the molecular weight (MW), theoretical isoelectric point (pI), grand average hydropathicity (GRAVY), and amino acid sequence length of the proteins. Gpos-mPLoc (http://www.csbio.sjtu.edu.cn/bioinf/Gpos-multi/.) and NPS-SOPMA (https://npsa-prabi.ibcp.fr/cgi-bin/secpred_sopma.pl) were used to predict the subcellular localization and secondary structure of MeGRAS proteins.

#### Phylogenetic, gene structure, and motif analysis of GRAS genes

Multiple sequence alignments of *Arabidopsis thaliana*, *M. truncatula*, and *M. edgeworthii* GRAS proteins were performed using MEGA 7.0.^30^ The exon-intron structures of the *MeGRASs* were visualized using TBtools software^27^ based on the GFF annotation file derived from the *M. edgeworthii* genome information. To analyze the conserved motif composition of *MeGRASs*, MEME (Multiple Em for Motif Elucidation) (http://meme-suite.org/index.html) was employed using default parameters.^31^ The search criteria were configured as follows: the optimal motif sequence width was set between 6 and 50 residues, with a maximum of 20 motifs identified, and other parameters were set to their default values. Each motif sequence was required to appear at least once. Additionally, conserved homologous domains encoded by *MeGRAS* gene were identified using MEGA 7.0^30^ and visualized with GeneDoc software.

#### Chromosomal location and analysis of the cis-regulatory elements in the promoter

Using the genome gff files of *M. edgeworthii*, TBtools software was used to perform chromosome localization analysis for each member of the GRAS gene family in *M. edgeworthii*. The structural features of MeGRAS proteins were analyzed using the corresponding annotation files in the genome database of *M. edgeworthii*. The types, quantities, and functions of cis-regulatory elements within the 2.0 kb promoter region upstream of the *MeGRAS* gene start codon were analyzed and visualized using the PlantCARE website (http://bioinformatics.psb.ugent.be/webtools/plantcare/html/).^32^ Additionally, the three-dimensional structures of MeGRAS proteins were predicted using the SWISS-MODEL (https://swissmodel.expasy.org/).^33^

#### Quantitative real-time reverse transcriptase-polymerase chain reaction (RTqPCR)

The RT-qPCR was performed on the Applied Biosystems 7500 PCR system using Fast Start Universal SYBR Green Master Mix (ROX). The reaction mixture was prepared as follows: 10.0 μL Fast Start Universal SYBR Green Master Mix, 0.6 μL each upstream and downstream primers (10 μM) (Table S2),^34^ 2.0 μL cDNA template, 6.8 μL nuclease-free H_2_O, resulting in a total reaction volume of 20 μL. The thermal cycling conditions were as follows: an initial step at 50°C for 120 s, followed by denaturation at 95°C for 10 min, and then 40 cycles of 95°C for 15 s, 60°C for 60 s, and 72°C for 34 s. The protocol concluded with a melting curve analysis: 95°C for 15 s, 60°C for 60 s, and 95°C for 15 s. All experiments were performed in three independent biological replicates. The 2^−ΔΔCT^ method was used to calculate the relative expression levels.^35^ To determine the statistical significance of the results, one-way analysis of variance (ANOVA) was performed using IBM SPSS Statistics version 2.0 software.

#### Subcellular localization

Primers were designed based on the coding DNA sequence of *MeGRASs* in *M. edgeworthii*. The complementary DNA (cDNA) obtained from total RNA was then used as a template to clone the target genes using the Phanta® Max Super-Fidelity DNA Polymerase gene amplification kit (Nanjing Novozymes Biotechnology Co., Ltd). The PCR reaction conditions were as follows: an initial denaturation at 94℃ for 3 min, followed by 32 cycles of denaturation at 94℃ for 15 s, annealing at 65℃ for 30 s, and extension at 72℃ for 1 min and 45 s. A final extension was performed at 72℃ for 10 min to ensure complete product synthesis. Following gel extraction and purification of the target fragments, the amplified genes were ligated into the pRI101-GFP vector via homologous recombination. The resulting recombinant plasmids were introduced into *Escherichia coli* DH5α competent cells using the heat shock transformation method. The transformed cells were plated on selective media containing the appropriate antibiotic. The following day, single colonies were isolated, and a small-scale culture was carried out to extract plasmids, which were then identified by sequencing. Upon confirmation of the correct plasmid, the construct was transformed into *Agrobacterium tumefaciens* strain EHA105, and successful transformation was verified by PCR. Subsequently, single colonies of *A. tumefaciens* were selected and cultured, and the recombinant bacteria were used for transient expression assays in *Nicotiana benthamiana* leaves via agroinfiltration. After 72 h, GFP expression in the infiltrated leaves was observed and documented under a laser confocal scanning microscope for subcellular localization analysis.

#### Transient expression analysis in tobacco leaves

To generate proDOF3.4:LUC, the promoter region of DOF3.4 was amplified using the primers proDOF3.4-F (5’-GCCAGTGCCAAGCTTCAAAAGAAAAATTTTATAC-3’) and proDOF3.4-R (3’-GTCTTCCATGTCGACGTTTAGTATTTGAAAGAAG-5’). The amplified fragments were cloned into pRI101 LUC vector to generate proDOF3.4:LUC construct. These constructs were then transformed into *Agrobacterium tumefaciens* strain EHA105. The reporter construct was co-infiltrated into tobacco leaves along with effector constructs expressing *35S:MeGRAS14, 35S:MeGRAS24, 35S:MeGRAS38, 35S:MeGRAS41, 35S:MeGRAS42*, or the corresponding empty vector control. After incubation for 3 d, the infiltrated leaves were treated with luciferin substrate and luminescence was detected using a CCD imaging system. All assays were performed with at least three independent biological replicates.

## Result

### Identification of GRAS family genes in M. edgeworthii

The availability of the complete *M. edgeworthii* genome enabled a comprehensive genome-wide identification of *GRAS* genes. To obtain an extensive overview of *GRAS* genes in *M. edgeworthii*, we conducted a genome-wide analysis. In the current study, we used a local BLAST search to compare the protein sequences of MtGRAS transcription factor family members from *Medicago truncatula* with the genome sequence of *M. edgeworthii*.^36^ As a result, 58 candidate *MeGRAS* genes were identified and named as *MeGRAS1-MeGRAS58*, respectively. To further investigate the physicochemical properties of the GRAS family sequence in *M. edgeworthii*, the online software Pfam^28^ and SMART database were used. The results obtained revealed considerable variation in the size of the full-length cDNA sequences of the *MeGRAS* genes, ranging from 636 bp (*MeGRAS58*) to 2448 bp (*MeGRAS46*). The full length of most GRAS proteins varied from 211 (MeGRAS58) to 815 (MeGRAS46) amino acids, with molecular weights ranging from 24.3 kDa (MeGRAS58) to 90.05 kDa (MeGRAS46). The theoretical isoelectric point (pI) ranged from 4.72 (MeGRAS50) to 9.91 (MeGRAS52). Additionally, 54 of the MeGRAS proteins had a theoretical isoelectric point (pI) <7, indicating that the majority of the MeGRAS proteins were rich in acidic amino acid residues. The calculated grand average of hydrophobicity (GRAVY) values for these proteins ranged from −0.69 (MeGRAS48) to 0.09 (MeGRAS58). Predictions of subcellular localization indicated that all members were localized in the nucleus except MeGRAS52, MeGRAS56, and MeGRAS58 (Table 1).

**Table 1.** The physicochemical properties of MeGRAS proteins.

Gene name	CDS length (bp)	Protein size (aa)	MW (kD)	PI	GRAVY
*MeGRAS1*	2190	729	81.27	5.38	−0.33
*MeGRAS2*	2196	731	81.44	5.38	−0.36
*MeGRAS3*	1779	592	65.83	4.81	−0.44
*MeGRAS4*	1713	570	64.33	5.06	−0.47
*MeGRAS5*	1449	482	54.53	5.80	−0.23
*MeGRAS6*	1461	486	55.62	7.53	−0.14
*MeGRAS7*	1386	461	52.74	5.69	−0.35
*MeGRAS8*	1443	480	53.99	5.57	−0.20
*MeGRAS9*	1791	596	66.88	5.20	−0.34
*MeGRAS10*	753	250	28.54	6.21	−0.19
*MeGRAS11*	1800	599	68.61	5.41	−0.30
*MeGRAS12*	1788	595	68.27	5.20	−0.33
*MeGRAS13*	960	319	36.63	5.88	−0.15
*MeGRAS14*	1740	579	64.46	5.72	−0.36
*MeGRAS15*	1377	458	51.69	5.42	−0.12
*MeGRAS16*	1968	655	74.44	5.45	−0.46
*MeGRAS17*	1869	622	71.36	5.45	−0.45
*MeGRAS18*	1926	641	72.60	5.38	−0.46
*MeGRAS19*	2205	734	83.10	5.53	−0.49
*MeGRAS20*	1971	656	74.46	5.15	−0.47
*MeGRAS21*	1971	656	74.46	5.69	−0.54
*MeGRAS22*	1356	451	51.90	5.71	−0.52
*MeGRAS23*	1218	405	46.06	5.88	−0.11
*MeGRAS24*	1629	542	61.87	5.23	−0.38
*MeGRAS25*	1536	511	56.56	5.62	−0.35
*MeGRAS26*	1923	640	73.21	5.46	−0.57
*MeGRAS27*	1923	640	73.21	5.46	−0.57
*MeGRAS28*	2142	713	81.41	5.38	−0.57
*MeGRAS29*	2046	681	77.31	5.44	−0.48
*MeGRAS30*	1968	655	73.64	5.84	−0.47
*MeGRAS31*	1359	452	50.04	5.36	−0.24
*MeGRAS32*	1419	472	53.53	6.64	−0.22
*MeGRAS33*	1689	562	62.38	5.09	−0.30
*MeGRAS34*	1335	444	50.04	5.67	−0.16
*MeGRAS35*	1515	504	57.70	4.97	−0.54
*MeGRAS36*	2031	676	75.11	5.48	−0.45
*MeGRAS37*	1566	521	58.67	6.24	−0.19
*MeGRAS38*	1632	543	60.96	5.82	−0.35
*MeGRAS39*	1485	494	55.96	5.28	−0.41
*MeGRAS40*	1374	457	51.60	5.69	−0.08
*MeGRAS41*	1599	532	60.03	5.17	−0.29
*MeGRAS42*	1635	544	61.03	5.18	−0.33
*MeGRAS43*	2028	675	74.85	5.59	−0.34
*MeGRAS44*	2232	743	83.96	6.18	−0.41
*MeGRAS45*	1326	411	49.97	5.27	−0.45
*MeGRAS46*	2448	815	90.05	6.10	−0.46
*MeGRAS47*	1671	556	64.57	6.00	−0.40
*MeGRAS48*	1665	554	61.67	5.92	−0.69
*MeGRAS49*	1626	541	60.31	5.57	−0.25
*MeGRAS50*	1524	507	58.38	4.72	−0.19
*MeGRAS51*	1443	480	54.52	4.76	−0.33
*MeGRAS52*	1551	516	58.59	9.91	0.02
*MeGRAS53*	864	287	31.77	4.78	−0.67
*MeGRAS54*	1281	426	48.15	5.74	−0.20
*MeGRAS55*	1623	540	59.75	5.44	−0.33
*MeGRAS56*	1560	519	58.80	9.47	0.01
*MeGRAS57*	1365	454	50.80	6.14	−0.56
*MeGRAS58*	636	211	24.30	8.83	0.09

Secondary structure analysis showed that MeGRAS proteins were composed of four different forms of secondary structures, which are dominated by α helices and irregular coiling, while β turn angles were the least abundant across all member species (Table S1). The subcellular localization prediction indicated that all members, except for MeGRAS52, MeGRAS56 and MeGRAS58 were localized in the nucleus (Table S1).

### Phylogenetic and structural analysis of MeGRAS genes

**Figure 1.**
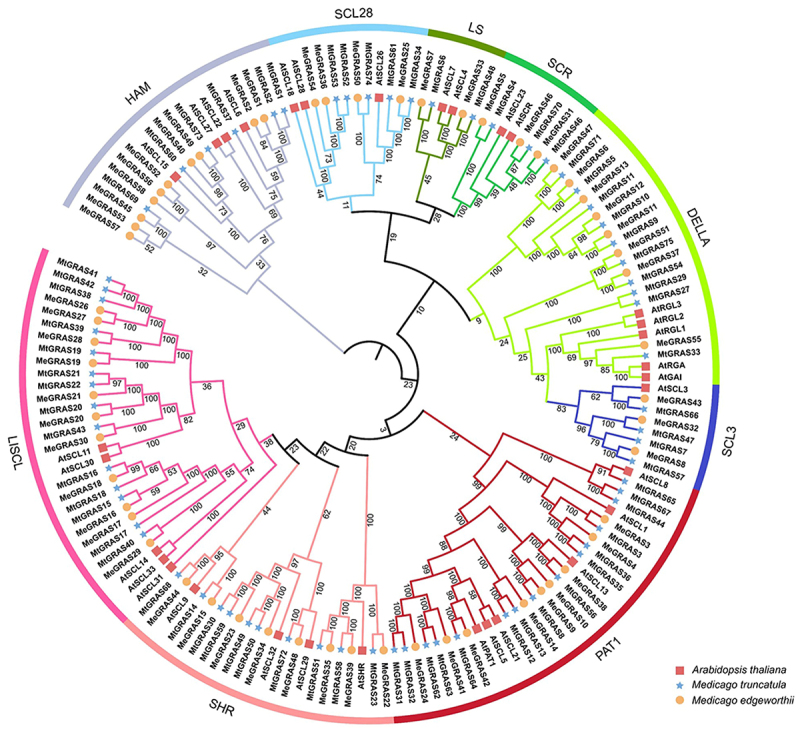
Phylogenetic tree of GRAS proteins from *M. edgeworthii*, *M. truncatula* and *Arabidopsis thaliana*. The unrooted phylogenetic tree was constructed by the Neighbor-Joining (NJ) method using MEGA 7.0 software incorporating 1000 bootstraps iterations. The analysis included 53 GRAS proteins, 32 from *Arabidopsis thaliana*, 68 from *M. truncatula*, and 58 from *M. edgeworthii*. These proteins were clustered into nine distinct subfamilies: LISCL, SHR, PAT1, SCL3, DELLA, SCR, LS, SCL28, and HAM, respectively.

To further evaluate the evolutionary relationships of GRAS proteins across different plant species, we selected the full-length amino acid sequences of 32 AtGRAS proteins from *A. thaliana*, 68 MtGRAS proteins from *Medicago truncatula*, and 58 MeGRAS proteins from *Medicago edgeworthii* for multiple sequence alignment and the subsequent construction of a Neighbour-Joining phylogenetic tree. Based on the established classification of GRAS proteins in *A. thaliana* and *M. truncatula*, the 58 MeGRAS proteins from *M. edgeworthii* were categorized into 9 subfamilies: LISCL, SHR, PAT1, SCL3, DELLA, SCR, LS, SCL28, and HAM. The distribution of protein across these subfamilies was as follows: 11 in LISCL, 8 in SHR, 9 in PAT1, 3 in SCL3, 8 in DELLA, 3 in SCR, 2 in LS, 4 in SCL28, and 10 in HAM, respectively (Figure 1). Notably, the PAT1 subfamily members were MeGRAS3, MeGRAS4, MeGRAS9, MeGRAS10, MeGRAS14, MeGRAS24, MeGRAS38, MeGRAS41 and MeGRAS42 (Figure 1). Using the MEME suite for motif prediction, we analyzed the *GRAS* family genes in *M. edgeworthii* to discern the relationships among GRAS transcription factors and the diversity within the GRAS protein family. The analysis revealed that GRAS proteins clustered within the same subfamily share similar motifs, with nearly all GRAS members containing motifs 4, 5, and 9, underscoring their conserved nature (Supplemental Figure 1). Moreover, the C-terminus region of MeGRAS proteins contained more motifs compared to the N-terminus region. Consequently, the C-terminus of GRAS proteins were more conserved, whereas the N-terminus exhibited greater variability. This variability in the N-terminal motifs contributes to the functional diversity of GRAS genes and might also explain the distinct functions observed among different subfamilies.

**Figure 2.**
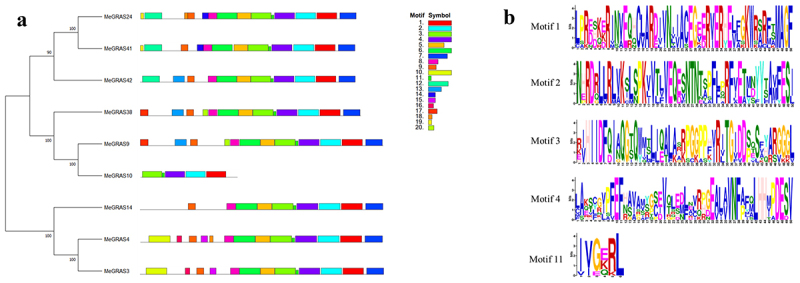
Phylogenetic and structural analysis of *PAT1* gene subfamily. (a) Phylogenetic tree and MEME protein structure of members of *PAT1* gene subfamily in *M. edgeworthii*. (b) Five most conserved motifs of *PAT1* gene subfamily in *M. edgeworthii*, with the bits representing the score of the site.

To confirm whether the screened genes were members of the GRAS family, multiple sequence alignment was performed using MEGA 7.0.^30^ The alignment results showed that all the screened genes possessed one or more GRAS conserved domains at the C-terminus (Supplemental Figure 2). Specifically, these domains included SAW, PFYRE, Leucine Heptad Repeat II (LHR II), VHIID, and Leucine Heptad Repeat I (LHR I), thereby confirming that the identified genes belong to the GRAS family (Supplemental Figure S2). To further investigate the structural diversity of members of the *PAT1* gene subfamily in *M. edgeworthii*, we identified and visualized the motif compositions of nine MeGRAS proteins using MEGA 7.0 and MEME software. A total of 20 conserved motifs were identified, labeled as motifs 1 to 20 (Figure 2a). Notably, motifs 1, 2, 3, 4, and 11 were present across most sequences, suggesting the functional similarity and conserved position among the *GRAS* genes in *M. edgeworthii* (Figure 2b).

### Chromosomal distributions of PAT1 subfamily genes

**Figure 3.**
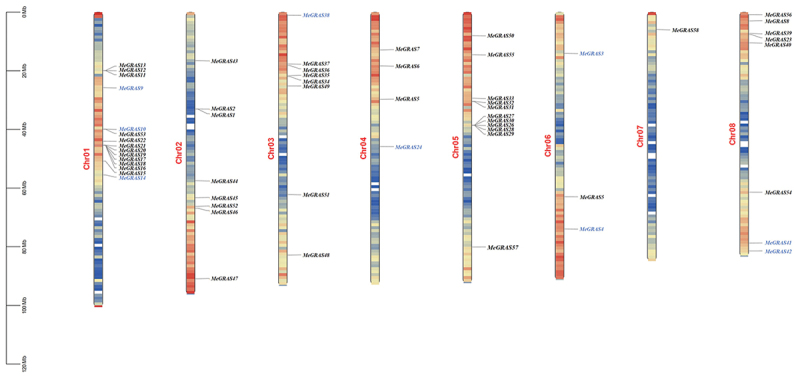
Distribution and localization of *MeGrass* gene family members on different chromosomes. Chromosome number is indicated at the left of each chromosome. Blue represents the *PAT1* subfamily genes.

Chromosomal localization analysis of the *GRAS* gene family members in *M. edgeworthii* was performed using TBtools software^27^ based on the available *M. edgeworthii* genome gff file. The 58 identified *MeGRAS* genes were unevenly distributed across the eight chromosomes of *M. edgeworthii*. Specifically, there were three genes on chromosome 6, four on chromosome 4, eight on each of chromosomes 2, 3 and 8. Additionally, 11 genes were clustered on chromosome 5, 15 on chromosome 1, and only one gene was located on chromosome 7 (Figure 3).

Analysis of the chromosomal localization of the nine *PAT1* subfamily genes according to the gene location files revealed that these genes were not distributed on chromosomes 2, 5 or 7 and were unevenly distributed across the remaining five chromosomes. Specifically, three genes (*MeGRAS9*, *MeGRAS10*, *MeGRAS14*) were located on chromosome 1, one gene (*MeGRAS38*) was located on chromosome 3, one gene (*MeGRAS24*) was located on chromosome 4, two genes (*MeGRAS3*, *MeGRAS4*) were located on chromosome 6, and two genes (*MeGRAS41*, *MeGRAS42*) were located on chromosome 8 (Figure 3).

### Cis-acting element analyses of PAT1 subfamily genes

**Figure 4.**
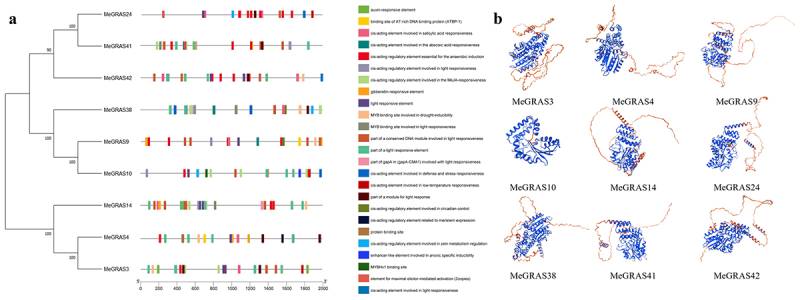
*Cis*-acting element analyses of *PAT1* subfamily genes. (a) *Cis*-acting elements of members of *PAT1* gene subfamily in *M. edgeworthii*. Promoter sequences (−2 kb) of 9 *MeGRAS* genes are analyzed by PlantCARE. Rectangles with different colors indicate different *cis*-acting elements participating in various abiotic stress regulations. (b) Three-dimensional conformation of members of *PAT1* gene subfamily in *M. edgeworthii*.

The *cis*-acting elements are crucial for the transcriptional regulation of gene expression. To further investigate the potential regulatory mechanism of the *PAT1* subfamily genes in *M. edgeworthii* under abiotic stress, the upstream 2.0 kb sequences of the *MeGRASs* gene were analyzed using the PlantCARE database for detecting the presence of responsive elements. The analysis identified 14 *cis*-acting elements related to responses to light, drought, low-temperature stress, plant hormone signaling pathway, and defense (Figure 4a). Furthermore, *cis*-acting elements exhibited variability among different genes. The majority of these elements were associated with light response, such as *cis*-acting regulatory elements involved in light response, MYB binding sites, and gapA-CMA1. This indicated the importance of these elements in regulating gene expression, enabling plants to participate in light signal transduction pathways. These findings further indicated that GRAS transcription factors are involved in several key processes related to the growth and development of *M. edgeworthii*, including light signal transduction, hormone signal transduction, and stress response.

The function of proteins is closely related to their structural conformation. The secondary structure of PAT1 subfamily proteins, including α helices, random coils, β turns, and elongation chains, is further intricately folded through the interaction of side chain groups, resulting in the formation of a compact spherical spatial structure maintained by various secondary bonds. To further analyze the protein structure, the online tool SWISS-MODEL was used to predict the three-dimensional conformation of the PAT1 subfamily proteins. Protein tertiary structure analysis of MeGRASs, with C-terminal conserved structural domains in blue and N-terminal variable structural domains in orange, revealed that the C-terminal structural domains were predominantly α-helical with β-folding, while the N-terminal structural domains were predominantly irregularly coiled. The structure of each protein has certain similarity, which in turn dictate its functional capabilities. The results indicated that all nine members of the *PAT1* gene subfamily exhibited similar three-dimensional structures (Figure 4b), suggesting that they may possess common functional characteristics.

### PAT1 subfamily genes expression analysis in M. edgeworthii

**Figure 5.**
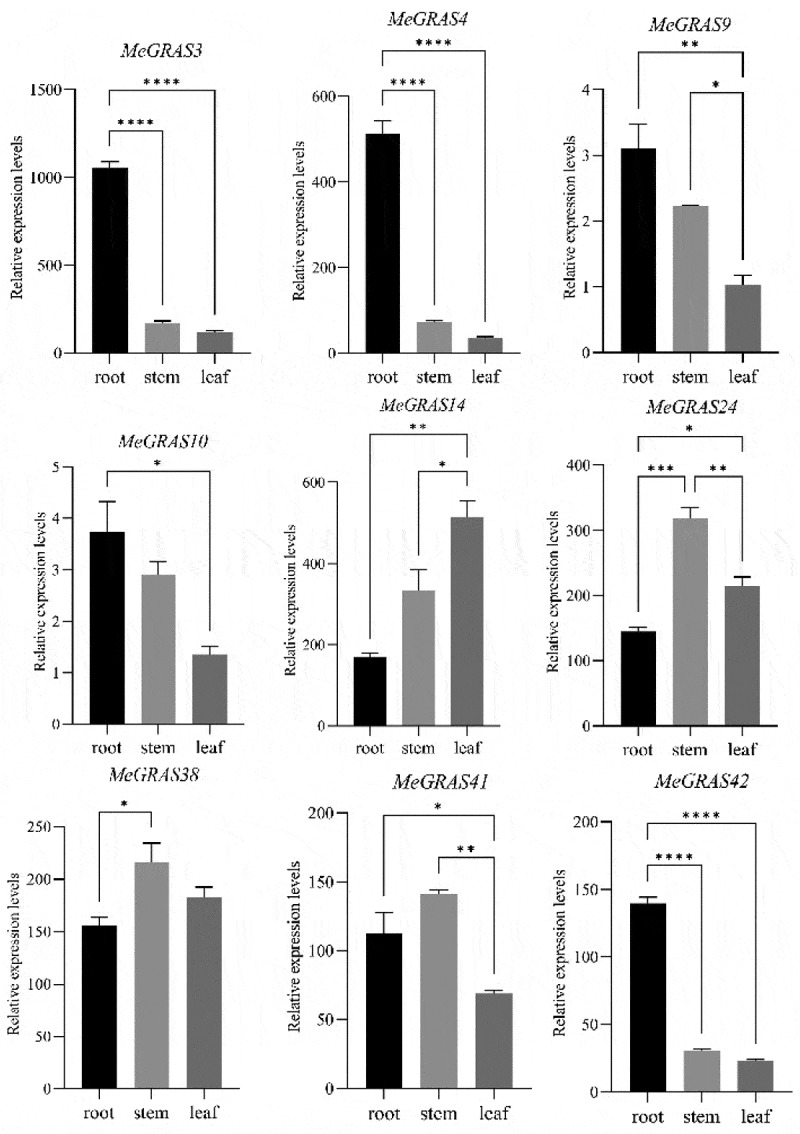
The relative expression levels of 9 *PAT1* genes in *M. edgeworthii* among different tissues. The error bars indicate standard deviation (SD), and the sample size was 3. The data are analyzed by one-way ANOVA (Duncan’s test). Asterisks above columns indicate the significance of a group: *p < 0.05, **p < 0.01, ***p < 0.001, ****p < 0.0001.

To assess the expression patterns of *PAT1* gene subfamily in *M. edgeworthii*, RT-qPCR analysis was conducted across three different tissues: roots, stems, and leaves. The results showed that all members of the *PAT1* gene subfamily were expressed in the roots, stems, and leaves. However, the expression levels of each gene varied across different tissue types, indicating their broad involvement in the development of various tissues (Figure 5). Comparative analysis of gene expression across tissues revealed that *MeGRAS14* had the highest expression level in leaves and shared the closest genetic relationship with Arabidopsis genes *AtSCL21* and *AtSCL5*. Additionally, the N-terminal of MeGRAS14 contained an EAISRRDL motif, which aligned with the previously reported functions of Arabidopsis *AtSCL21* and *AtSCL5* in regulating the phytochrome signaling pathway.^14^ In contrast, *MeGRAS24* and *MeGRAS38* had the highest expression levels in stem. Phylogenetic analysis revealed that *MeGRAS38* shared the closest phylogenetic relationship with *AtSCL13*, suggesting that *MeGRAS38* mainly plays a role in the elongation zone of the hypocotyl.^15^ Furthermore, *MeGRAS3*, *MeGRAS4*, *MeGRAS9*, *MeGRAS10*, and *MeGRAS42* showed the highest expression levels in roots, providing further evidence for the involvement of PAT1 subfamily genes in plant root growth and development.^20,21^

### Cloning and subcellular localization analysis of partially PAT1 subfamily genes in M. edgeworthii

**Figure 6.**
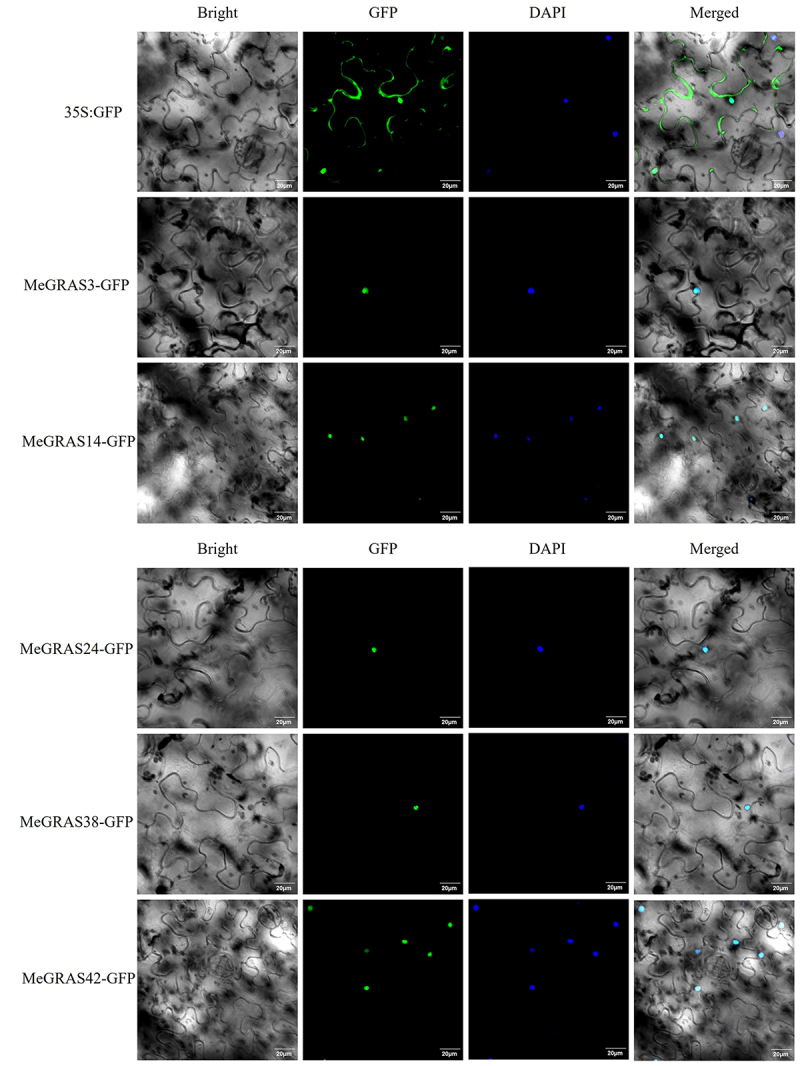
Fluorescence map of the subcellular localization of MeGRAS3, MeGRAS14, MeGRAS24, MeGRAS38 and MeGRAS42. 35S: GFP: expression of 35S-GFP/pRI101-GFP empty vector in tobacco leaves. Scale bar = 20 μm.

Determining a protein’s subcellular localization is important for elucidating its functional role within the cell. For this purpose, specific primers were designed for *MeGRAS3*, *MeGRAS14*, *MeGRAS24*, *MeGRAS38*, and *MeGRAS42*, for which preliminary insights into their biological functions were already available. PCR amplification was performed using cDNA from *M. edgeworthii* to clone these genes. The coding sequences (CDS) of the five *MeGRASs* were then ligated into the pRI101-GFP vector and transferred into Agrobacterium for subsequent transient expression in tobacco leaves. Confocal microscopy analysis revealed that all five MeGRAS proteins were located on the nucleus (Figure 6).

### The PAT1 subfamily genes activate the expression of the MeDOF3.4 gene in M. edgeworthii

**Figure 7.**
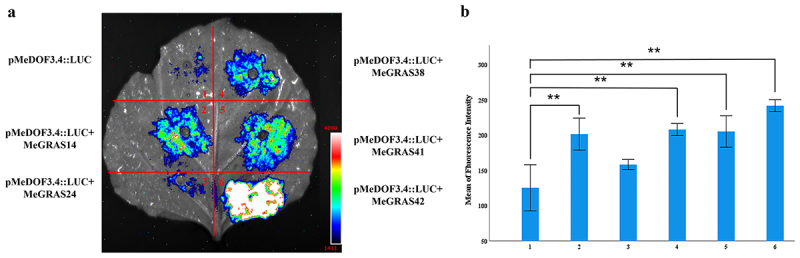
The *PAT1* subfamily genes activate the expression of the *MeDOF3*.4 gene in *M. edgeworthii*. Transient expression assays (a) and the mean of fluorescence intensity (b) confirmed that MeGRAS14, MeGRAS24, MeGRAS38, MeGRAS41, and MeGRAS42 can activate the expression of the *MeDOF3.4* gene. The error bars indicate standard deviation (SD), and the sample size was 3. The data are analyzed by one-way ANOVA (Duncan’s test). Asterisks above columns indicate the significance of a group: **p < 0.01. “1” indicates “pMedof3.4:luc”; “2” indicates “pMedof3.4:luc + MeGRAS14”; “3” indicates “pMedof3.4:luc + MeGRAS24”; “4” indicates “pMedof3.4:luc + MeGRAS38”; “5” indicates “pMedof3.4:luc + MeGRAS41”; “6” indicates “pMedof3.4:luc + MeGRAS42”.

The PAT1 subfamily functions as a transcription factor by binding to the promoters of DNA binding with one finger (Dof) family genes, thereby promoting cell proliferation and tissue regeneration.^23^ A previous report claimed that SCL5/SCL21/PAT1 can activate the expression of the *DOF3.4* gene.^19^ To investigate whether similar transcriptional activation exists in *M. edgeworthii*, we constructed a proDOF3.4: LUC reporter gene line by fusing its promoter (2000 kb) to the LUC gene. Transient expression assays in tobacco leaves confirmed that MeGRAS14, MeGRAS38, MeGRAS41, and MeGRAS42 can activate the expression of the *MeDOF3.4* gene (Figure 7a,b). These results suggested that the PAT1 subfamily might perform a similar function in both *Arabidopsis* and *M. edgeworthii*.

## Discussion

GRAS, as unique transcription factor in plants, plays an important regulatory role in several biological processes such as plant growth and development, hormone and light signaling, as well as abiotic stress response.^12^ However, to date, no comprehensive analysis of this gene family has been conducted in *Medicago edgeworthii*.

This study identified and characterized 58 members of the *MeGRAS* gene family from the entire genome of *M. edgeworthii*. All MeGRAS proteins showed significant structural diversity, indicating a high level of complexity of this gene family. In this study, the amino acid length of the proteins encoded by the *GRAS* gene family members in *M. edgeworthii* varied from 211 to 815 amino acids, which was comparable to the variability observed in other plant species such as maize,^37^ soybean,^38^ and *Eucalyptus grandis*.^39^ Similarly, the isoelectric points (pI) of these proteins varied from 4.72 to 9.91, with 54 proteins having an isoelectric point below 7 and 4 proteins having an isoelectric point above 7, suggesting a predominance of acidic proteins. These results were consistent with those reported for soybean^38^ and sweet potato,^40^ indicating that the physicochemical properties of *GRAS* gene family members were conserved across different species. The *GRAS* genes of *M. edgeworthii* were categorized into nine subfamilies. GRAS proteins clustered within the same subfamily had similar motifs, suggesting that members of the same subfamily may perform similar functions.

Due to the close relationship between the structure and properties of proteins and their functions, this study analyzed the protein properties and structural characteristics of the *GRAS* gene family in *M. edgeworthii*. By comparing the full-length sequence of amino acids, it was found that the C-terminus of the GRAS protein amino acid sequence in *M. edgeworthii* had high conservation, characterized by the presence of five conserved motifs: LHRI/II, VHIID, PYRE, and SAW. In contrast, the N-terminal sequences of these amino acid sequences had significant differences, and the predicted results of the protein’s tertiary structure were also consistent. Subcellular localization software predicted the localization of GRAS protein in the nucleus. Subcellular localization experiments in tobacco leaves revealed the, nuclear localization, which was consistent with the results of the analysis software.

Members of the *Arabidopsis PAT1* gene subfamily are involved in plant life activities such as defense against stress, elongation of hypocotyl, light signal response pathways,^11^ and cell fusion reactions.^20^ Structural analysis and examination of conserved protein domains of the *M. edgeworthii PAT1* gene subfamily revealed that among the 25 predicted *cis*-acting elements identified in the 9 *PAT1* gene subfamily members, 14 were related to plant hormones, light, drought stress, low temperature, defense, and stress responses. These findings were consistent with existing research reports. The *MeGRAS3*, *MeGRAS24*, and *MeGRAS14* genes had high expression levels in roots, stems, and leaves, respectively. It was speculated that these genes mainly participate in growth and development of roots and hypocotyls, as well as to participate in light response signal transduction pathways. Previous studies have shown that the *PAT1* gene subfamily is a conserved regulator of graft healing and tissue regeneration,^20^ while the *DOF3.4* gene is a key factor driving cell division during plant regeneration.^19^ Notably, *Arabidopsis* SCL5/SCL21/PAT1 have been shown to activate the expression of *DOF3.4* gene, which promotes cell division and tissue regeneration.^19^ In this study, tobacco transient expression experiments showed that several PAT1 subfamily proteins in *M. edgeworthii* can also activate the expression of *MeDOF3.4* gene. Further explanation suggests that the PAT1 subfamily may have similar functions in *Arabidopsis* and *M. edgeworthii*.

In conclusion, using TBtools software,^27^ we discovered and characterized nine members of the *M. edgeworthii PAT1* gene subfamily. A comprehensive analysis was conducted into their phylogenetic relationships, gene structures, expression profiles across various tissues, and subcellular localizations. Our results suggested that these nine *PAT1* subfamily genes may play a role in defense against stress response, hypocotyl growth and development, light signal response pathways, and cell fusion response. Overall, the genomic identification and bioinformatics analysis of the *M. edgeworthii PAT1* gene subfamily provide a reference for further elucidation of the functions of MeGRAS family genes in *M. edgeworthii*, and provide promising candidate genes for the molecular breeding and genetic improvement of *M. edgeworthii*.

## Supplementary Material

Supplemental Material

## Data Availability

The data that support the findings of this study are available on request from the corresponding author.
