# Supplementary material for: Identification and characterization of nine PAT1 genes subfamily in Medicago edgeworthii
Source: Plant Signal Behav. 2025 Jul 1;20(1):2527380. doi: 10.1080/15592324.2025.2527380 (PMC12218496; doi:10.1080/15592324.2025.2527380)
Supplement: Supplemental Material [file KPSB_A_2527380_SM8480.docx]

# Supplementary Figures and Tables

## Supplementary Figures


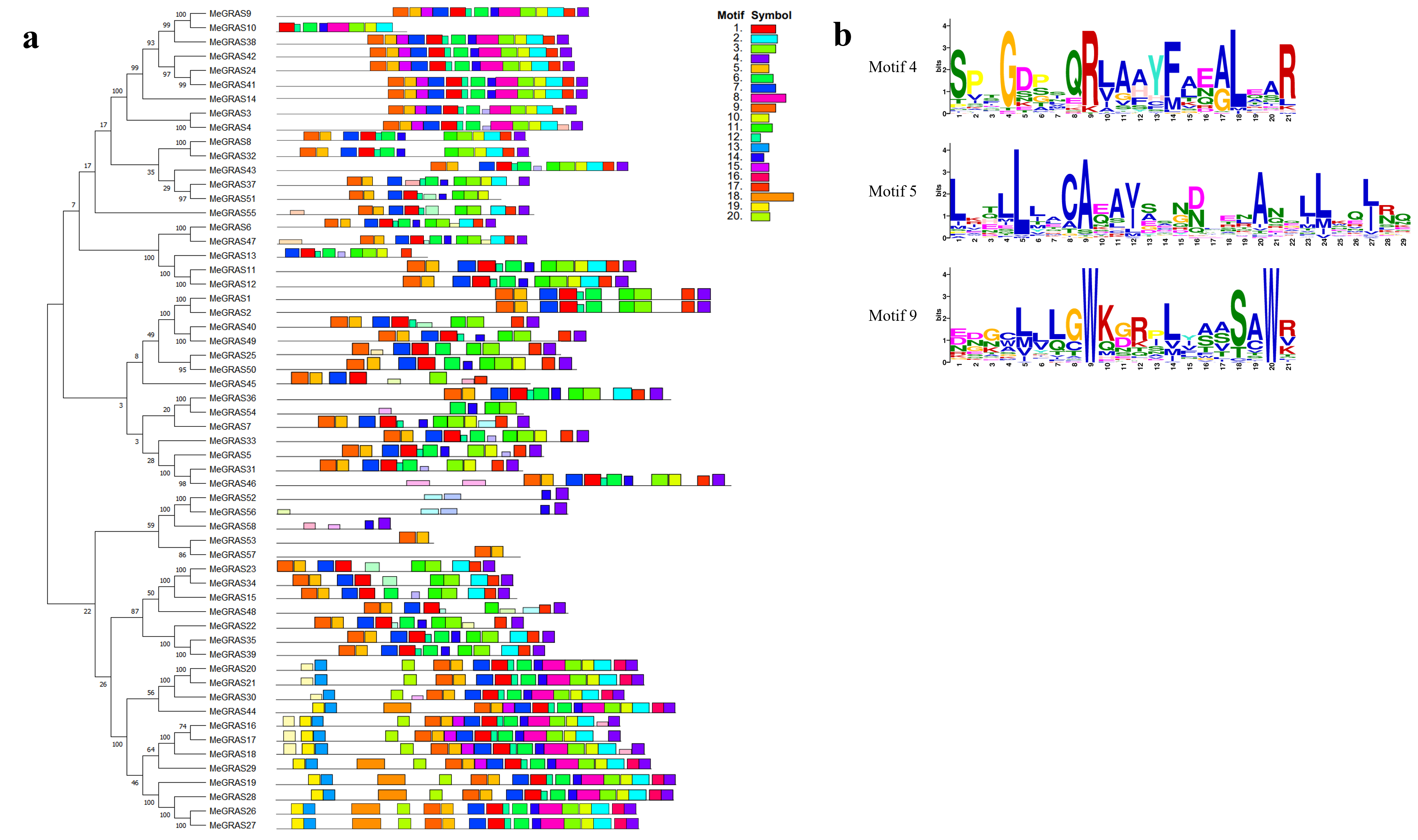


**Supplemental Figure 1** Phylogenetic and structural analysis of *MeGRASs* gene family. **a** The phylogenetic tree of GRAS proteins in *M. edgeworthii* and structural map of MEME proteins. **b** Three most conserved motifs of MeGRAS proteins, and the bits represent the score of the site.


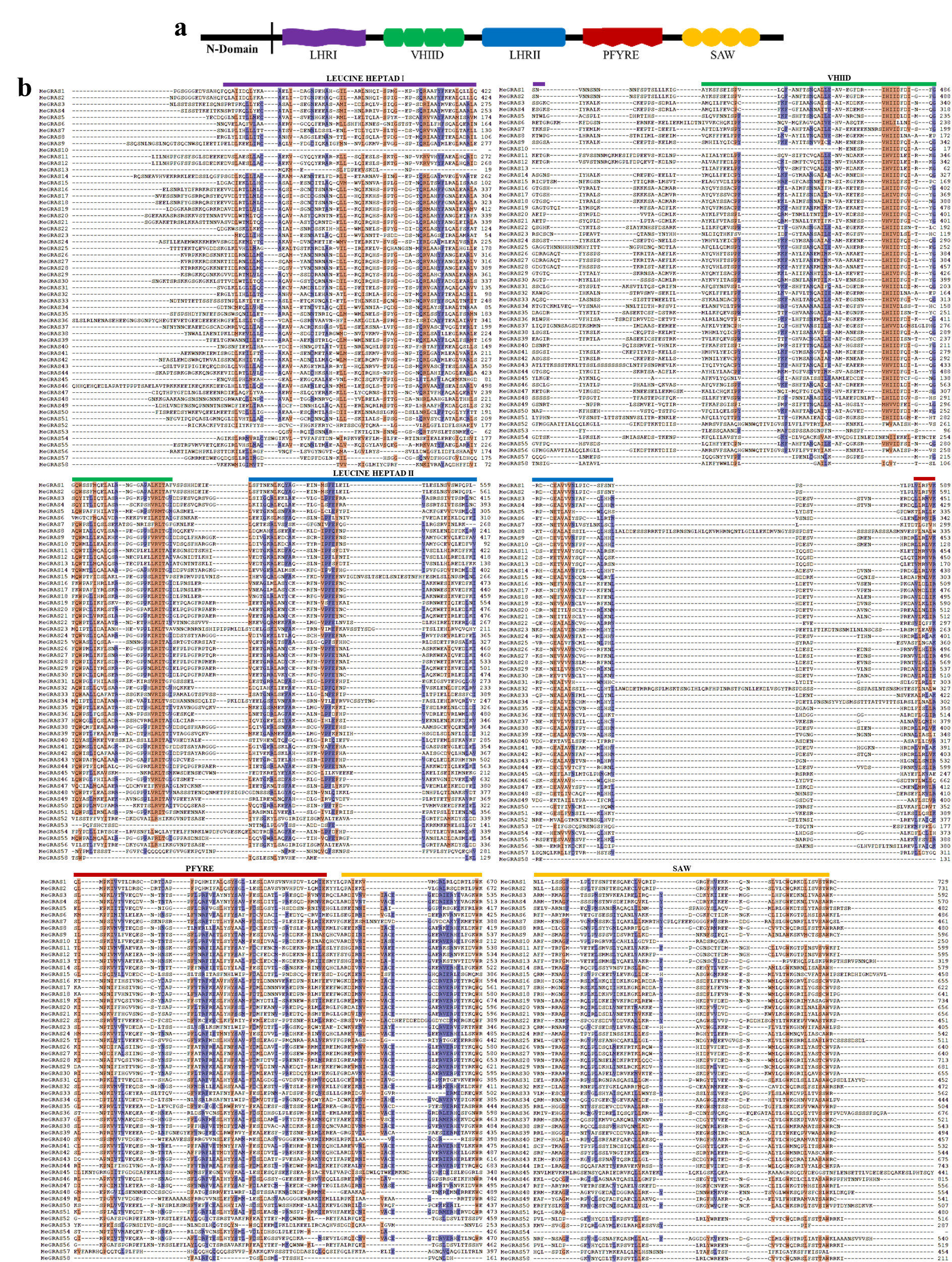


**Supplemental Figure 2** Alignment of MeGRAS proteins sequences. **a** Structural features of a typical GRAS protein. The GRAS domain comprises of five motifs designated as, LHRI/II, VHIID, PYRE, and SAW. **b** MeGRAS proteins homologous sequence alignment.

## Supplementary Tables

Table S1 The secondary structure and subcellular localization of MeGRAS proteins

| Protein name | α-helix/No. | Extend strand/No. | β turn/No. | Random coil/No. | Subcellular localization |
| --- | --- | --- | --- | --- | --- |
| MeGRAS1 | 240 | 69 | 22 | 398 | nucleus |
| MeGRAS2 | 233 | 73 | 25 | 400 | nucleus |
| MeGRAS3 | 248 | 70 | 26 | 248 | nucleus |
| MeGRAS4 | 222 | 70 | 29 | 249 | nucleus |
| MeGRAS5 | 211 | 48 | 22 | 201 | nucleus |
| MeGRAS6 | 260 | 56 | 25 | 145 | nucleus |
| MeGRAS7 | 240 | 52 | 24 | 145 | nucleus |
| MeGRAS8 | 242 | 56 | 20 | 162 | nucleus |
| MeGRAS9 | 265 | 46 | 29 | 256 | nucleus |
| MeGRAS10 | 127 | 31 | 18 | 74 | nucleus |
| MeGRAS11 | 288 | 59 | 23 | 229 | nucleus |
| MeGRAS12 | 305 | 54 | 24 | 212 | nucleus |
| MeGRAS13 | 167 | 42 | 20 | 90 | nucleus |
| MeGRAS14 | 230 | 56 | 25 | 268 | nucleus |
| MeGRAS15 | 215 | 56 | 28 | 159 | nucleus |
| MeGRAS16 | 303 | 83 | 29 | 240 | nucleus |
| MeGRAS17 | 259 | 60 | 24 | 243 | nucleus |
| MeGRAS18 | 269 | 73 | 23 | 276 | nucleus |
| MeGRAS19 | 292 | 70 | 33 | 339 | nucleus |
| MeGRAS20 | 291 | 62 | 30 | 273 | nucleus |
| MeGRAS21 | 285 | 50 | 29 | 292 | nucleus |
| MeGRAS22 | 202 | 57 | 22 | 170 | nucleus |
| MeGRAS23 | 203 | 45 | 25 | 132 | nucleus |
| MeGRAS24 | 250 | 54 | 23 | 215 | nucleus |
| MeGRAS25 | 220 | 55 | 20 | 216 | nucleus |
| MeGRAS26 | 299 | 53 | 23 | 265 | nucleus |
| MeGRAS27 | 286 | 55 | 26 | 273 | nucleus |
| MeGRAS28 | 300 | 61 | 27 | 325 | nucleus |
| MeGRAS29 | 286 | 63 | 30 | 302 | nucleus |
| MeGRAS30 | 279 | 57 | 25 | 293 | nucleus |
| MeGRAS31 | 207 | 46 | 22 | 177 | nucleus |
| MeGRAS32 | 241 | 45 | 21 | 165 | nucleus |
| MeGRAS33 | 210 | 71 | 22 | 259 | nucleus |
| MeGRAS34 | 209 | 41 | 24 | 170 | nucleus |
| MeGRAS35 | 225 | 79 | 25 | 175 | nucleus |
| MeGRAS36 | 237 | 63 | 27 | 349 | nucleus |
| MeGRAS37 | 254 | 56 | 25 | 186 | nucleus |
| MeGRAS38 | 235 | 57 | 23 | 228 | nucleus |
| MeGRAS39 | 216 | 49 | 48 | 230 | nucleus |
| MeGRAS40 | 212 | 61 | 16 | 168 | nucleus |
| MeGRAS41 | 250 | 48 | 24 | 210 | nucleus |
| MeGRAS42 | 245 | 49 | 23 | 227 | nucleus |
| MeGRAS43 | 271 | 58 | 24 | 322 | nucleus |
| MeGRAS44 | 298 | 72 | 29 | 344 | nucleus |
| MeGRAS45 | 254 | 45 | 25 | 117 | nucleus |
| MeGRAS46 | 296 | 122 | 43 | 354 | nucleus |
| MeGRAS47 | 292 | 57 | 25 | 182 | nucleus |
| MeGRAS48 | 215 | 58 | 28 | 253 | nucleus |
| MeGRAS49 | 223 | 67 | 19 | 232 | nucleus |
| MeGRAS50 | 281 | 55 | 13 | 158 | nucleus |
| MeGRAS51 | 260 | 37 | 13 | 170 | nucleus |
| MeGRAS52 | 220 | 111 | 57 | 128 | cell membrane |
| MeGRAS53 | 95 | 34 | 6 | 152 | nucleus |
| MeGRAS54 | 173 | 426 | 33 | 163 | nucleus |
| MeGRAS55 | 283 | 48 | 26 | 183 | nucleus |
| MeGRAS56 | 236 | 105 | 55 | 123 | cell membrane |
| MeGRAS57 | 87 | 39 | 12 | 316 | nucleus |
| MeGRAS58 | 113 | 39 | 16 | 43 | vacuoles |

Table S2 The RT-qPCR primers for *M. edgeworthii*

| Gene name | Forward primer (5´-3´) | Reverse primer (5´-3´) |
| --- | --- | --- |
| *Me-Actin* | TGGTGTCATGGTTGGTATGG | CTCTGTTGGCCTTTGGGTTA |
| *MeGRAS3* | GAAGGAAATGAAGCAAAAGCC | TCCCCGATGAAGCCAAAC |
| *MeGRAS4* | CCCTGATTTGTCCTCCAAGA | GGACTGATCAGCACCACGTT |
| *MeGRAS9* | TGTGAAGAACCAACAAGCAAAG | AGCCTCCAAAAGTAACATCCA |
| *MeGRAS10* | TTTTGGAGGCTCTCAAGCAT | CCAGTTGAACCTCACATCCA |
| *MeGRAS14* | TCCTTGAAAGTTGGCTTTGG | TGGCGTATAATGGTTGGTTTC |
| *MeGRAS24* | TCAACACCAATAACGCTCCG | CCACTCTTTCTTCCCCTTCG |
| *MeGRAS38* | TGCAAATGTCTCAGCAAAGG | ATTCCAGGGTGAAGTGTTGC |
| *MeGRAS41* | AGCATGTTCAGCATTGATCC | GCTTCGAGACCGGACTATTG |
| *MeGRAS42* | GAGGCCAAGCAACTCAATTC | CTTGCTGCGACATTGGACTA |
